# A live attenuated RHΔ*ompdc*Δ*uprt* mutant of *Toxoplasma gondii* induces strong protective immunity against toxoplasmosis in mice and cats

**DOI:** 10.1186/s40249-023-01109-9

**Published:** 2023-06-15

**Authors:** Yu Shen, Bin Zheng, Hao Sun, Songrui Wu, Jiyuan Fan, Jianzu Ding, Meng Gao, Qingming Kong, Di Lou, Haojie Ding, Xunhui Zhuo, Shaohong Lu

**Affiliations:** 1 School of Basic Medical Sciences and Forensic Medicine, Hangzhou Medical College, Hangzhou, Zhejiang China; 2 Engineering Research Center of Novel Vaccine of Zhejiang Province, Hangzhou Medical College, Hangzhou, China; 3 Key Laboratory of Bio-Tech Vaccine of Zhejiang Province, Hangzhou Medical College, Hangzhou, China

**Keywords:** *Toxoplasma gondii*, Orotidine-5'-monophosphate decarboxylase, Phosphoribosyltransferase, Live attenuated vaccine, Immunization, Mouse, Cat

## Abstract

**Background:**

*Toxoplasma gondii* is an obligate intracellular apicomplexan parasite and is responsible for zoonotic toxoplasmosis. It is essential to develop an effective anti-*T. gondii* vaccine for the control of toxoplasmosis, and this study is to explore the immunoprotective effects of a live attenuated vaccine in mice and cats.

**Methods:**

First, the *ompdc* and *uprt* genes of *T. gondii* were deleted through the CRISPR-Cas9 system. Then, the intracellular proliferation and virulence of this mutant strain were evaluated. Subsequently, the immune responses induced by this mutant in mice and cats were detected, including antibody titers, cytokine levels, and subsets of T lymphocytes. Finally, the immunoprotective effects were evaluated by challenge with tachyzoites of different strains in mice or cysts of the ME49 strain in cats. Furthermore, to discover the effective immune element against toxoplasmosis, passive immunizations were carried out. GraphPad Prism software was used to conduct the log-rank (Mantel–Cox) test, Student’s *t* test and one-way ANOVA.

**Results:**

The RHΔ*ompdc*Δ*uprt* were constructed by the CRISPR-Cas9 system. Compared with the wild-type strain, the mutant notably reduced proliferation (*P* < 0.05). In addition, the mutant exhibited virulence attenuation in both murine (BALB/c and BALB/c-nu) and cat models. Notably, limited pathological changes were found in tissues from RHΔ*ompdc*Δ*uprt-*injected mice. Furthermore, compared with nonimmunized group, high levels of IgG (IgG1 and IgG2a) antibodies and cytokines (IFN-γ, IL-4, IL-10, IL-2 and IL-12) in mice were detected by the mutant (*P* < 0.05). Remarkably, all RHΔ*ompdc*Δ*uprt*-vaccinated mice survived a lethal challenge with RHΔ*ku80* and ME49 and WH6 strains. The immunized sera and splenocytes, especially CD8^+^ T cells, could significantly extend (*P* < 0.05) the survival time of mice challenged with the RHΔ*ku80* strain compared with naïve mice. In addition, compared with nonimmunized cats, cats immunized with the mutant produced high levels of antibodies and cytokines (*P* < 0.05), and notably decreased the shedding numbers of oocysts in feces (95.3%).

**Conclusions:**

The avirulent RHΔ*ompdc*Δ*uprt* strain can provide strong anti-*T. gondii* immune responses, and is a promising candidate for developing a safe and effective live attenuated vaccine.

**Graphical abstract:**

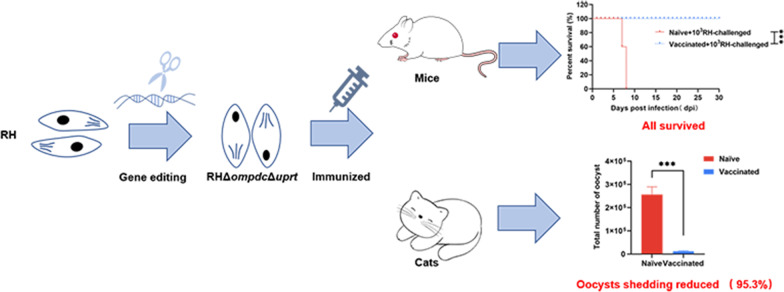

**Supplementary Information:**

The online version contains supplementary material available at 10.1186/s40249-023-01109-9.

## Background

*Toxoplasma gondii* is an intracellular protozoan parasite and can infect nearly all warm-blooded animals including humans [1]. It is estimated that approximately one-third of the world's population is infected with *T. gondii* [2]. Humans acquire *T. gondii* infection usually through ingestion of tissue cysts in raw or undercooked meat, oocysts in food or water, or congenitally via the placenta [3, 4]. Although *T. gondii* usually appears as a latent infection in people with normal immunity, it can cause serious complications in individuals with suppressed immune systems such as AIDS patients or people with malignant tumors [5, 6]. Pregnant women infected with *T. gondii* may experience miscarriage and stillbirth, and the fetus will have deformities or severe intellectual disability after birth [7]. The infection of intermediate animals including cattle, sheep and pigs may result in huge economic losses to the farm and potential health risks to humans [8].

Prevention or treatment of toxoplasmosis is difficult due to the complex life cycle and elaborate immune escape system of the parasite to establish chronic infection in most hosts [3, 9]. So far, toxoplasmosis is mainly treated with drugs such as pyrimethamine and sulfadiazine. However, these drug treatments are only effective in the acute infection stage and may cause serious side effects and promote the development of drug-resistant strains [10–12]. Therefore, novel drugs and effective treatments for *T. gondii* infection should be discovered and developed.

Vaccination is a promising and effective long-term approach for disease control and prevention [13]. The current anti-toxoplasmosis vaccines mainly include protein vaccine, DNA vaccine, live vector vaccine and live attenuated vaccine [13]. Among the existing vaccines against *T. gondii*, live attenuated vaccines provided the best protection with robust cellular and humoral responses to toxoplasmosis in murine models [14]. Moreover, the only commercial *T. gondii* vaccine (Toxovax®) is also a live attenuated vaccine developed through continuous subculture from S48 *T. gondii* tachyzoites [15, 16]. However, due to the potential risk of virulence recovery, the vaccine can’t be used in humans and is mainly used in sheep. In contrast, genetic deletions may completely prevent virulence regression compared to naturally attenuated strains [7]. Recently, several studies have demonstrated the stability and protective effect of gene knockout live attenuated vaccines against *T. gondii*. For example, Li et al. developed a double gene deletion mutant of *gra17* and *npt1* in the RH strain of *T. gondii*, which can protect mice from acute, chronic and congenital toxoplasmosis [17].

Pyrimidine plays an important role in parasite proliferation. Thus, as the precursor of all pyrimidines, uridine monophosphate (UMP) is one of the most important nucleotides in RNA for *T. gondii* [18, 19]. Through de novo biosynthesis or salvage pathways, *T. gondii* can acquire adequate pyrimidine and UMP for propagation [20]. The de novo pyrimidine biosynthesis pathway mainly functions under normal circumstances, while the salvage pathway only functions when the de novo biosynthesis pathway is blocked [21]. Studies have shown that when the de novo pyrimidine biosynthesis pathway of *T. gondii* is disrupted, it has little effect on the ability of *T. gondii* to invade host cells, but its proliferation and virulence are significantly weakened [20]. Orotidine-5'-monophosphate decarboxylase (OMPDC) is the terminal enzyme belonging to the de novo biosynthesis pathway, and studies have found that after knocking out the *ompdc* gene of *T. gondii*, the mutant strain lost its replication ability and virulence [22, 23]. According to these studies, the pyrimidine auxotrophic strain of *T. gondii* can be used as an alternative live attenuated vaccine. However, we cannot ignore the fact that *T. gondii* with *ompdc* gene knockout can still proliferate weakly in vitro [24]. As mentioned above, uracil phosphoribosyltransferase (UPRT) is a key enzyme in the UMP salvage pathway [25], which could contribute to parasite proliferation when the de novo biosynthesis pathway is blocked. Thus, to completely block the uracil synthesis pathway, here we generated a mutant with double gene deletion of *ompdc* and *uprt* in the RHΔ*ku80*Δ*hxgprt* strain by a clustered regularly interspaced short palindromic repeats (CRISPR)/cas9 system and evaluated the immune protection of this strain against toxoplasmosis.

## Methods

### Animals and ethics statement

Six- to eight-week-old BALB/c and BALB/c-nu mice were purchased from the Center of Laboratory Animal of Hangzhou Medical College and 3-month-old cats were purchased from a local breeder. All cats were tested serologically and found to be free of *T. gondii* and viruses, including feline calicivirus, coronavirus, feline immunodeficiency virus, feline leukemia virus, and feline parvovirus. All animals were raised under standard conditions according to the Animal Management Regulations of the People's Republic of China. Animal experiments were approved by the Animal Care and Use Committee of Hangzhou Medical College (2018–027).

### Parasite and cell culture

Tachyzoites of the ME49, WH6, RHΔ*ku80*Δ*hxgprt*, RHΔ*ku80*Δ*uprt*::HXGPRT and RHΔ*ku80*Δ*uprt*Δ*hxgprt* strains were maintained in human foreskin fibroblasts (HFFs) in our laboratory in high-glucose Dulbecco’s Modified Eagle Medium (DMEM; Gibco, Thermo Fisher Scientific, MA, USA) supplemented with 5% fetal bovine serum (FBS; Gibco, Thermo Fisher Scientific, MA, USA), penicillin (100 units/ml; Thermo Fisher Scientific, MA, USA), and streptomycin (100 µg/ml; Thermo Fisher Scientific, MA, USA). The RHΔ*ku80*Δ*uprt*Δ*ompdc*::HXGPRT strain was additionally treated with 250 μmol/L uracil and 200 μmol/L UMP (Sigma-Aldrich, MO, USA) [20]. HFFs were cultured in high-glucose DMEM supplemented with 10% FBS at 37 °C, and 5% CO_2_. In all experiments, freshly egressed tachyzoites were filtered with 5-µm polycarbonate membranes to remove host cell debris.

### Preparation of soluble *T. gondii* antigens (STAg)

Soluble *T. gondii* antigens were prepared as previously described [26]. In brief, suspensions of *T. gondii* RHΔ*ku80* tachyzoites were collected in phosphate buffered saline (PBS), subjected to repeated freeze and thaw cycles, and then sonicated on ice at 80 W/s. The prepared product was centrifuged at 14,000 × *g* for 30 min at 4 °C. The supernatant was filtered through 0.22 μm sterile nitrocellulose filters. The STAg concentration was determined by the Bradford kit (Beyotime, Shanghai, China), and aliquots were stored at − 80 °C until use.

### Construction of RHΔ*uprt* and RHΔ*ompdc*Δ*uprt* mutant strains

The primers involved in this experiment are listed in Additional file 1: Table S1. The mutant strains were constructed using the CRISPR/Cas9 approach based on the RHΔ*ku80*Δ*hxgprt* strain according to Shen B’s protocol [27]. Briefly, the *hxgprt* and *ompdc* targeting CRISPR plasmids were generated by replacing the *uprt* targeting guide RNA in pSAG1::CAS9-U6::sgUPRT with an *hxgprt* and *ompdc* single-guide RNA by site-directed mutagenesis (New England Biolabs, MA, USA). All plasmids were verified by DNA sequencing prior to use. First, the *uprt* CRISPR plasmid and HXGPRT homologous template were electro transfected into RHΔ*ku80*Δ*hxgprt* tachyzoites. The *uprt*-deleted parasites were screened with 25 μg/ml xanthine (Sigma-Aldrich, MO, USA) and 25 μg/ml mycophenolic acid (Sigma-Aldrich, MO, USA), and single-cloned by limiting the dilution. RHΔ*ku80*Δ*uprt*::HXGPRT single positive clones were identified by PCR and qRT-PCR. Second, the *hxgprt* CRISPR plasmid and *uprt*5’UTR-3’UTR homologous template were electro transfected into RHΔ*ku80*Δ*uprt*::HXGPRT tachyzoites. The RHΔ*ku80*Δ*uprt*Δ*hxgprt* strain was screened with 10 μmol/L 5-fluorodeoxyuracil (Sigma-Aldrich, MO, USA) and identified by PCR. Finally, the *ompdc* CRISPR plasmid and HXGPRT homologous template were electro transfected into RHΔ*ku80*Δ*uprt*Δ*hxgprt* tachyzoites. The RHΔ*ku80*Δ*uprt*Δ*ompdc*::HXGPRT strain was screened and identified in the same way as described above. The PCRs were carried out in a volume of 25 μl containing 12.5 μl of 2 × *Taq* PCR Master Mix (TIANGEN, Beijing, China), 1 μl of each primer (10 µmol/L), 1 μl genomic DNA template, and 9.5 μl of sterile distilled water by the conditions with an initial melting step at 98 ℃ for 3 min, followed by 30 cycles with each cycle at 98 ℃ for 30 s, 60 ℃ for 30 s, and 72 ℃ for 1 min, followed by a final extension at 72 ℃ for 10 min.

Total RNA of *T. gondii* tachyzoites was extracted using the TRIzol reagent (Invitrogen, CA, USA). The cDNA was synthesized using a First Strand cDNA Synthesis Kit (ReverTra Ace -α-, Toyobo, Osaka, Japan). qRT-PCR was carried out in a volume of 20 μl containing 10 μl of 2 × Real time PCR Master Mix (Toyobo, Osaka, Japan), 0.4 μl of each primer (10 µmol/L), 1 μl cDNA template, and 8.2 μl of sterile distilled water and amplification was performed on a CFX96 Touch™ Real-Time PCR Detection System (Bio-Rad, CA, USA). The relative mRNA levels were calculated using the comparative ΔCt method using the formula 2^−ΔΔCt^.

### Parasite intracellular replication assay

An indirect immunofluorescence assay (IFA) was used to detect the intracellular proliferation of parasites. Equal amounts of RHΔ*ku80*Δ*ompdc*Δ*uprt* and RHΔ*ku80* strains were inoculated in plates filled with HFF. The wells inoculated with RHΔ*ku80*Δ*ompdc*Δ*uprt* strains were cultured with or without 250 μmol/L uracil and 200 μmol/L UMP for 24 h and 48 h, and the cells were fixed with 4% paraformaldehyde (Beyotime, Shanghai, China) solution for 30 min. The cells were incubated with rabbit anti-GRA7 polyclonal antibody and Alexa Fluor488-conjugated goat anti-rabbit IgG (Abcam, Oxford, UK). The intracellular parasites at different stages of proliferation (i.e., 1, 2, 4, 8, 16 or more than 16 tachyzoites) were counted from 100 parasitophorous vacuoles under fluorescence microscopy (Nikon eclipse 80i, Tokyo, Japan). The experiment was performed in three independent biological repeats.

### Parasite plaque assay

HFF monolayers grown in six-well plates were infected with 500 tachyzoites of RHΔ*ku80* or RHΔ*ku80*Δ*ompdc*Δ*uprt* strains in each well. RHΔ*ku80*Δ*ompdc*Δ*uprt* strains were cultured with or without 250 μmol/L uracil and 200 μmol/L UMP. After 7 days, the cells were fixed with 4% paraformaldehyde (Beyotime, Shanghai, China) and stained with crystal violet. Finally, the number and size of plaques were analyzed.

### Evaluation of the infectivity of the mutant parasites in mice

Tachyzoites of the RHΔ*ku80* and RHΔ*ku80*Δ*ompdc*Δ*uprt* strains were washed and resuspended in PBS. Four groups of mice were injected intraperitoneally (i.p.) with 0.1 ml of PBS, 1 × 10^2^ tachyzoites of the RHΔ*ku80* strain or 1 × 10^5^ or 1 × 10^6^ tachyzoites of the RHΔ*ku80*Δ*ompdc*Δ*uprt* strain (5 BALB/c mice per group). Moreover, serial doses (1 × 10^2^, 1 × 10^3^, 1 × 10^4^, 1 × 10^5^, 1 × 10^6^ tachyzoites) of the RHΔ*ompdc*Δ*uprt* strain or 1 × 10^2^ tachyzoites of RHΔ*ku80* strain were i.p. injected into immunodeficient mice (5 BALAB/c-nu mice per group). Survival of mice was monitored daily for 30 days.

### Evaluation of the infectivity of the mutant parasites in cats

Two groups of cats were injected intramuscularly (i.m.) with 0.1 ml of PBS or 1 × 10^7^ tachyzoites of the RHΔ*ku80*Δ*ompdc*Δ*uprt* strain, faecal samples were collected daily from 1 day post infection (dpi) to 10 dpi and monitored for *T. gondii* oocysts. The oocysts were purified as described previously [28]. Briefly, first, 1 g of feces was weighed and mixed with an appropriate amount of water and centrifuged to collect the sediment. Next, the sediment was mixed with 10 times the volume of sucrose solution with a specific gravity of 1.15 and centrifuged (1500 × *g*). Finally, approximately 5 ml of the supernatant was mixed with 45 ml of water and centrifuged (1500 × *g*); the sediment was resuspended in 1 ml of water and counted under a light microscope (Nikon Ti-S, Tokyo, Japan).

### Detection of parasite load

Six- to eight-week-old BALB/c mice (36 per group) were infected i.p. with 1 × 10^6^ tachyzoites of the RHΔ*ku80* and RHΔ*ku80*Δ*ompdc*Δ*uprt* strains. The ascites of mice (30 per group) were collected from day 1 to day 5, and mice (6 per group) were executed on the fourth day to collect liver and lung tissues. Genomic DNA was sacrificed by using the DNeasy Blood & Tissue Kit (Qiagen, Hilden, Germany). Amplifications of genomic DNA were carried out with primers targeting the repeated 529 bp gene of *T. gondii*. The qPCR system was as described above and the amplification was performed on a CFX96 Touch™ Real-Time PCR Detection System (Bio-Rad, CA, USA). The parasite burden was subsequently determined through a standard curve of the 529 bp gene.

### Immunization and challenge in mice

Mice (30 per group) were intraperitoneally immunized with 1 × 10^6^/100 µl RHΔ*ku80*Δ*ompdc*Δ*uprt* strain tachyzoites or an equivalent amount of PBS, once every two weeks, for a total of three immunizations. Mouse sera were collected from the tail vein at 0, 2, 4, and 6 weeks. After centrifugation at 4000 × *g* for 5 min, the sera were collected and stored at − 20 °C until further use. Two weeks after the last immunization, mice were intraperitoneally injected with 1 × 10^3^ tachyzoites of the RHΔ*ku80* strain, 1 × 10^3^ bradyzoites of the ME49 strain, or 1 × 10^3^ tachyzoites of the WH6, and the survival rate was recorded daily.

### Immunization and challenge in cats

Cats (6 per group) were intramuscularly immunized with 1 × 10^7^/100 µl RHΔ*ku80*Δ*ompdc*Δ*uprt* strain tachyzoites or an equivalent amount of PBS once every three weeks for a total of two immunizations. Sera samples were collected at 0, 2 and 4 weeks and stored at − 20 °C until further use. One week after the last immunization, oral administration of 200 ME49 cysts was carried out in immunized and control cats. According to the above method, feces were collected and purified for oocyst counting for 15 consecutive days after the challenge.

### Measurement of antibody responses in mice and cats

Antibody levels of IgG, IgG1, and IgG2a of mice or IgG of cats were detected by enzyme-linked immunosorbent assay (ELISA). Briefly, 96-well microtiter plates were coated with 100 μl (10 µg/ml) STAg (diluted in PBS) and incubated at 4 °C overnight. Then the plates were washed five times with PBS containing 0.05% Tween 20 (PBST) and blocked with PBST containing 5% non-fat milk powder for 1 h at 37 °C. The plates were washed five times with PBST. Then, 100 µl of sera samples diluted in PBST (1:100) containing 5% non-fat milk powder were added to the wells and incubated at 37 °C for 1 h. The plates were washed five times with PBST, then 100 µl of diluted horseradish-peroxidase-conjugated goat anti-mouse IgG (Abcam, Oxford, UK, 1:10,000), anti-mouse IgG1 (Abcam, Oxford, UK, 1:5000), IgG2a (Abcam, Oxford, UK, 1:5000) or horseradish-peroxidase-conjugated goat anti-cat IgG (Abcam, Oxford, UK, 1:5000) was added for incubation for 1 h at 37 °C. After washing five times, 100 µl of 3,3′,5,5′-tetramethylbenzidine (TMB) chromogen solution (Beyotime, Shanghai, China) was added to each well and the plates were incubated at 37 °C for 15 min. After adding 100 µl of stop solution for TMB substrate (Beyotime, Shanghai, China), the absorbance was measured by an ELISA plate reader at 450 nm.

### Cytokine assay

Mice (5 per group) were sacrificed two weeks after the last immunization, and the spleens were aseptically removed to prepare a single-cell suspension. Briefly, the spleen was placed on a 70 μm cell filter mesh and then added into a 50 ml centrifuge tube by using a 5 ml syringe plunger to grind the spleen. Hank's solution was added dropwise while grinding, and the mesh was rinsed with Hank's solution after grinding to obtain a single cell suspension. The supernatant was discarded after centrifugation, and then 5 times the cell volume erythrocyte lysing solution was added to the cell. Afterwards, the mixture was gently mixed by pipetting and lysed for 2 min. After centrifugation again, the supernatant was discarded, and the cells were resuspended in high-glucose DMEM containing 20% FBS and counted on a bovine abalone counter. Splenocytes (1 × 10^6^) from different groups of mice were seeded in sterile 96-well cell culture plates with a final volume of 100 µl. The culture supernatant was added to STAg at a final concentration of 10 μg/ml and the supernatant was collected for 24 h, 72 h, 96 h. The levels of secreted interleukin-2 (IL-2), interleukin-4 (IL-4), interleukin-10 (IL-10), interleukin-12 (IL-12) and interferon-γ (IFN-γ) were measured by flow cytometry (BD Biosciences, Franklin Lakes, NJ, USA) using a BD™ Cytometric Bead Array (CBA) kit (BD Biosciences, Franklin Lakes, NJ, USA).

### Lymphocyte proliferation assay

Two weeks after the last immunization, the spleens of three mice from each group were prepared and resuspended as described above. Splenocytes (1 × 10^5^) from different groups of mice were added to 96-wellplates and stimulated with STAg (10 µg/ml) or DMEM high-glucose medium of equal volume (negative control). Moreover, no cells were added, and only the wells of medium were added as a blank control. Then, the splenic lymphocytes were incubated at 37 °C for 96 h with 5% CO_2_. Cell Counting Kit 8 (CCK-8, Solarbio, Beijing, China) (10 µl) was added according to the instructions and incubated for 4 h. Subsequently, the absorbance was measured by an ELISA plate reader at 450 nm to illustrate lymphocyte proliferation. The cell proliferation activity was calculated using the following formula: cell proliferation activity = (OD_450_ STAg−OD_450_ Blank)/(OD_450_ Control−OD_450_ Blank).

### Flow cytometry analysis of T cell subsets

To analyze the percentage of CD4^+^ and CD8^+^ T lymphocytes, 1 × 10^6^ splenocytes were prepared as described above and suspended in 100 µl PBS. After incubation with fluorochrome-labelled mAbs including FITC-CD3, APC-Cy7-CD4 and PE-CD8 (BD Biosciences, Franklin Lakes, NJ, USA) at room temperature for 15 min in the dark, the cultures were washed with 2 ml PBS. After centrifugation, the samples were suspended in 500 µl PBS and fluorescence profiles were analyzed on a flow cytometer (BD Biosciences, Franklin Lakes, NJ, USA) by FlowJo software (BD Biosciences, Franklin Lakes, NJ, USA, version 10.8.1).

### Analysis of hematoxylin–eosin (HE) staining of the liver, spleen, and lung

The liver, spleen, and lung tissues of mice from each group (3 mice per group) were removed and soaked in 4% paraformaldehyde (Beyotime, Shanghai, China) at room temperature for 24 h, dehydrated with ethanol, cleared with xylene, embedded in wax and sliced with a slicer. Sections were then stained with hematoxylin and eosin (H&E) as described previously [29].

### Passive immunization of sera and splenocytes from RHΔ*ompdc*Δ*uprt* -vaccinated mice

Splenocyte suspensions without erythrocytes were prepared as described above, CD19^+^ B cells, CD8^+^ T cells or CD4^+^ T cells were purified (> 90% purity) using Miltenyi Mouse positive selection kits (Miltenyi Biotec, Cologne, Germany). Naïve mice received splenocytes (1 × 10^7^), CD19^+^ B cells (2 × 10^6^), CD4^+^ T cells (2 × 10^6^) or CD8^+^T cells (2 × 10^6^) from RHΔ*ku80*Δ*ompdc*Δ*uprt*-vaccinated mice, or splenocytes (1 × 10^7^) from naïve mice via tail vein injection. After cell transfer for 24 h, mice were intraperitoneally injected with 1 × 10^3^ tachyzoites of the RHΔ*ku80* strain (5 mice per group) [30]. Two weeks after the last immunization, sera from immunized mice were collected as positive sera. In addition, sera from naïve mice were collected as negative sera. Subsequently, BALB/c mice (5 per group) were i.p. challenged with 1 × 10^3^ RHΔ*ku80* tachyzoites, and injected with positive or negative sera (200 μl/mouse) via the tail vein from day 0 to day 4. Survival of mice was monitored daily and the parasitic load in mouse (5 per group) peritoneal fluid was detected one day after the end of sera treatment.

### Statistical analyses

Statistical analyses in this study were performed with GraphPad Prism version 8 (GraphPad Software Inc, CA, USA). The log-rank (Mantel–Cox) test was used to compare the survival curves. Independent Student’s t-test was conducted to compare two groups and one-way ANOVA was conducted to compare ≥ 3 groups. Quantitative variables were presented as the means ± standard deviations (SD). In all analyses, *P* < 0.05 was considered statistically significant.

## Results

### Generation of the RHΔ*ompdc*Δ*uprt* Strains Using the CRISPR/Cas9 System

**Fig. 1:**
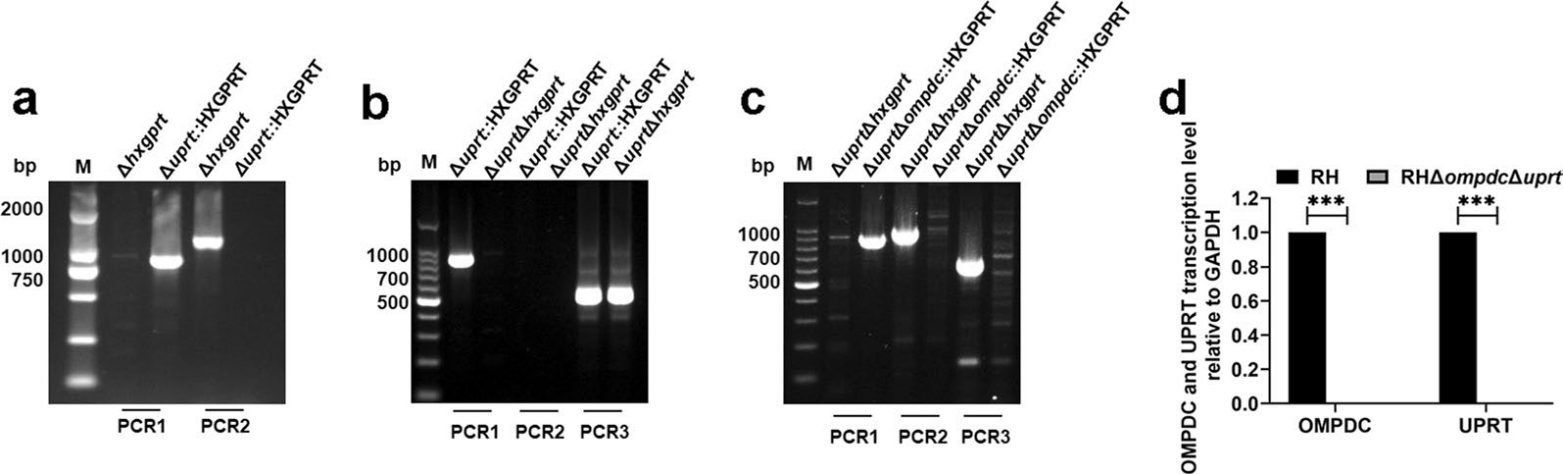
CRISPR/Cas9-mediated generation of the *ompdc-uprt* deletion mutant in the *Toxoplasma gondii* type I RH strain. **a** Diagnostic PCR of the RHΔ*uprt*::HXGPRT clone. PCR1 is used to detect drug gene and PCR2 is used to detect *uprt* gene **b** Diagnostic PCR confirming the removal of *hxgprt*. PCR1 is used to detect *hxgprt* gene, PCR2 is used to detect *uprt* gene and PCR3 is used to detect internal reference 529 bp gene **c** Diagnostic PCR of the RHΔ*uprt*Δ*ompdc*::HXGPRT clone. PCR1 is used to detect drug gene, PCR2 and PCR3 is used to detect *ompdc* gene **d** qRT-PCR of the RHΔ*uprt*Δ*ompdc*::HXGPRT clone. ***P < 0.001 by Student’s *t* test. *CRISPR* clustered regularly interspaced short palindromic repeats, *PCR* polymerase chain reaction, *HXGPRT* hypoxanthine–guanine phosphoribosyltransferase, *qRT-PCR* quantitative reverse transcription polymerase chain reaction

The HXGPRT marker was inserted into the *uprt*-specific guide RNA-targeted sequence region **(**Additional file 1: Fig. S1a**)**. The single and stable RHΔ*ku80*Δ*uprt*::HXGPRT clone was verified by PCR detection (Fig. 1a). Under the effects of sgRNA of *hxgprt* and enzyme Cas9, the HXGPRT marker was deleted (Additional file 1: Fig. S1b), and PCR results indicated that a single RHΔ*ku80*Δ*uprt*Δ*hxgprt* strain was successfully selected (Fig. 1b). Subsequently, the *ompdc* gene was replaced by the HXGPRT marker in the RHΔ*ku80*Δ*uprt*Δ*hxgprt* strain to yield the RHΔ*ompdc*Δ*uprt* mutant (Additional file 1: Fig. S1c), and the stable mutant clone was determined by PCR and qPCR assays (Fig. 1c, d). In summary, the double gene knockout RHΔ*ku80*Δ*uprt*Δ*hxgprt* strain was successfully generated.

### The RHΔ*ompdc*Δ*uprt* mutant exhibited reduced cellular replication ability

**Fig. 2:**
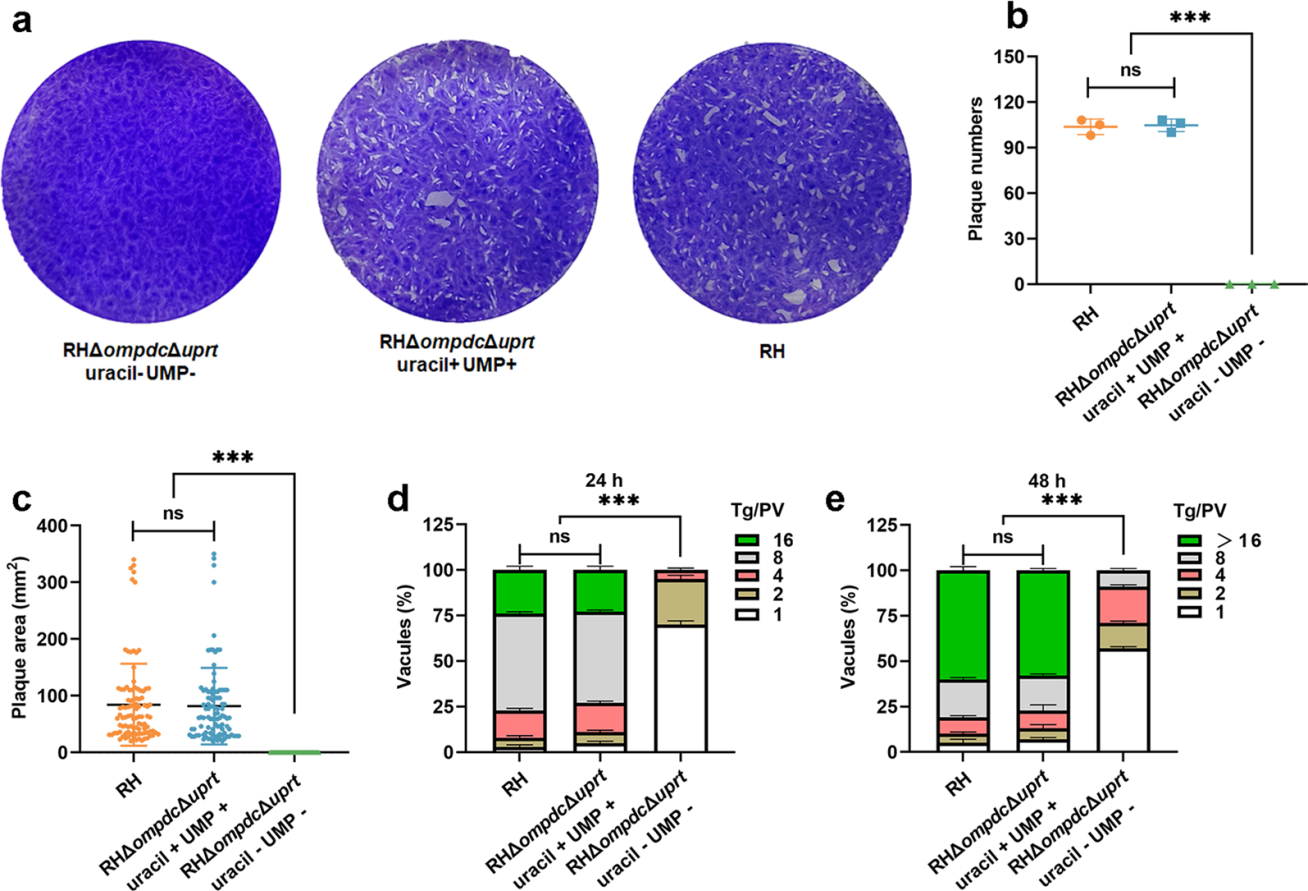
Deficiency of *ompdc* and *uprt* in the RH strain severely reduced parasitic proliferation in vitro. **a** Plaque assay comparing the growth of the RHΔ*ku80* strain and RHΔ*ku80*Δ*ompdc*Δ*uprt* strain with or without the addition of 250 µmol/L uracil and 200 µmol/L UMP. **b**–**c** Number and size of the plaques. **d**–**e** Intracellular proliferation of the RHΔ*ku80* strain and RHΔ*ku80*Δ*ompdc*Δ*uprt* strain with or without the addition of 250 µmol/L uracil and 200 µmol/L UMP. The number of parasites in each parasitophorous vacuole (PV) was determined at 24 h and 48 h. These results are from three independent trials. ***P < 0.001 by Student’s *t* test and one-way ANOVA, ns not significant. *UMP* uridine monophosphate, *PV* parasitophorous vacuole

To explore the biological characteristics of the RHΔ*ompdc*Δ*uprt* mutant, invasion, parasite replication, and plaque assays were carried out. Plaque assay results showed that in the presence of uracil and ump, the mutant could grow as normally as the wild type RHΔ*ku80* strain to form plagues, while plagues were rarely observed when the uracil and ump were removed from the culture medium of the RHΔ*ompdc*Δ*uprt* mutant (Fig. 2a–c). In addition, the parasite replication assay showed that at 24 h and 48 h post infection, the number of RHΔ*ompdc*Δ*uprt* mutant tachyzoites per parasitophorous vacuole (PV) was notably lower (*P* < 0.05) than that of wild type RHΔ*ku80* tachyzoites in the absence of uracil and ump, and most PVs of this mutant consisted of 1 or 2 tachyzoites indicating a nearly quiescent condition of cellular proliferation (Fig. 2d, e, Additional file 1: Fig. S2). However, the number of RHΔ*ompdc*Δ*uprt* mutant tachyzoites per PV presented a similar level to that of the wild type once supplied with uracil and ump, indicating the replication restoration. Furthermore, the results of the invasion assay showed limited differences between the wild type strain and the RHΔ*ompdc*Δ*uprt* mutant regardless of whether they were supplied with or without uracil and ump (Additional file 1: Fig. S3), suggesting that the deletion of *ompdc* and *uprt* had no effect on invasion.

### The virulence of the RHΔ*ompdc*Δ*uprt* mutant was severely attenuated in mice

**Fig. 3:**
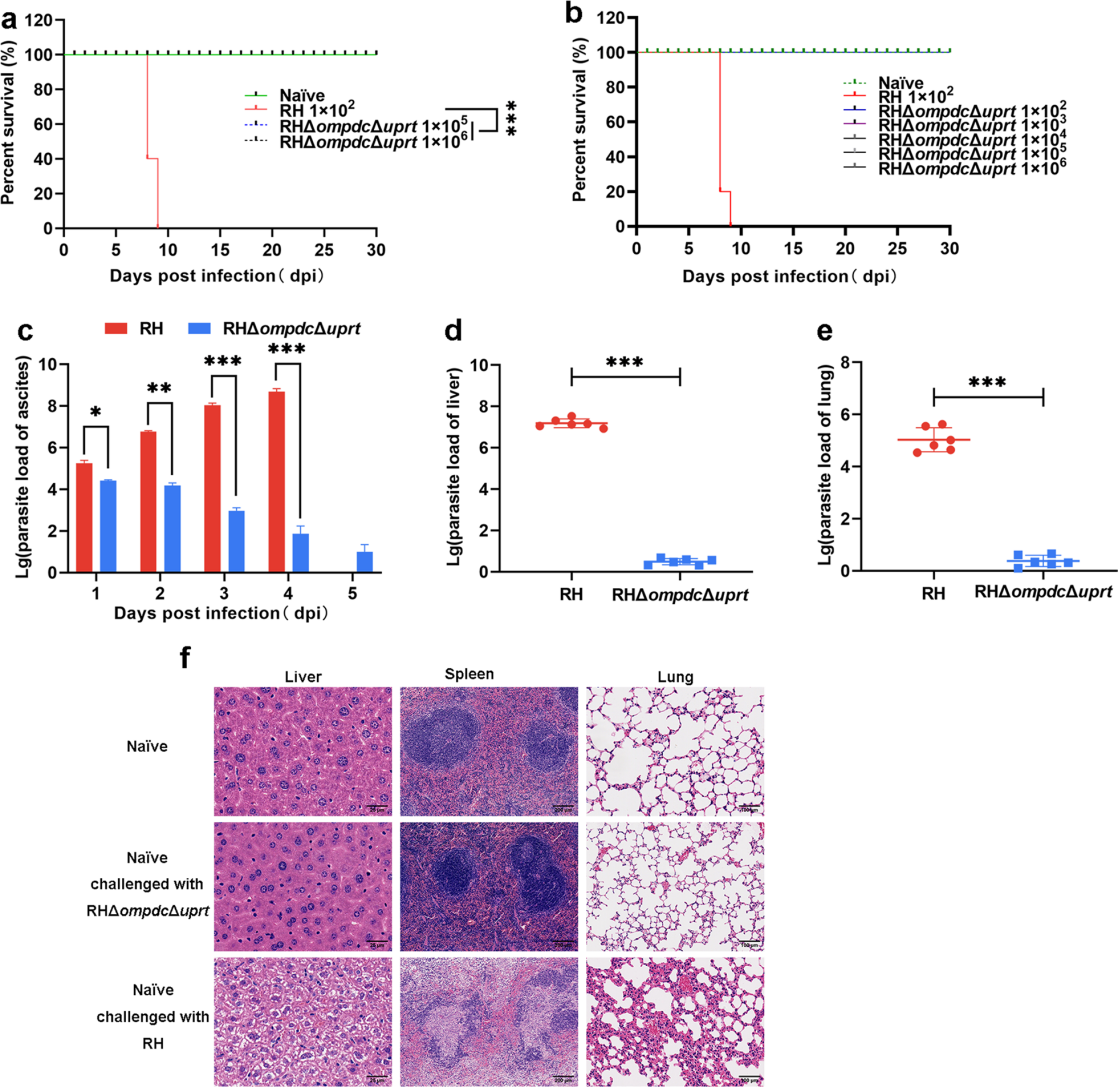
The virulence of the RHΔ*ku80*Δ*ompdc*Δ*uprt* mutant was severely attenuated in mice. **a** Tachyzoites of RHΔ*ku80*Δ*ompdc*Δ*uprt* or RHΔ*ku80* tachyzoites were injected intraperitoneally (i.p.) into BALB/c mice (*n* = 5) and monitored for more than 30 d. **b** Tachyzoites of RHΔ*ku80*Δ*ompdc*Δ*uprt* or RHΔ*ku80* tachyzoites were injected i.p. into BALB/c-nu mice (*n* = 5) and monitored for more than 30 d. **c** The parasite numbers in ascitic fluid of BALB/C mice infected with the 1 × 10^6^ RHΔ*ku80*Δ*ompdc*Δ*uprt* strain and RHΔ*ku80* strain from 1 to 5 days. **d**–**e** The parasite burden of liver and lung tissues infected with the RHΔ*ku80*Δ*ompdc*Δ*uprt* strain and RHΔ*ku80* strain for 4 days. **f** Tissue damage in the spleen, liver and lung of naïve mice inoculated with the RHΔ*ku80* strain and defective strain respectively. The 529 bp gene was detected by qPCR to demonstrate the number of *T. gondii* tachyzoites in each sample. ***P < 0.001 by Student’s t test and log-rank (Mantel–Cox) test. *i.p.* intraperitoneally, *T. gondii Toxoplasma gondii, qPCR* quantitative polymerase chain reaction

The survival of mice was monitored daily, and the results showed that mice injected with the wild-type strain all died within 9 days, while the survival rates of RHΔ*ompdc*Δ*uprt* mutant-injected mice were 100%, even when challenged with an infectious high dose of 10^6^ tachyzoites (Fig. 3a, b), indicating that the virulence of this mutant was significantly attenuated. Subsequently, ascites samples from different groups of mice were collected from 1 to 5 dpi for parasite examination. The parasite number in wild type-infected mice increased remarkably day by day, while that of RHΔ*ompdc*Δ*uprt*-injected mice notably decreased (*P* < 0.05) every day (Fig. 3c). Similarly, a large number of *T. gondii* tachyzoites were detected in the liver and lung tissues of RHΔ*ku80*-injected mice, while limited tachyzoites were detected by qPCR in these tissues from RHΔ*ompdc*Δ*uprt*-injected mice on the 4th day post infection (Fig. 3d, e). These results demonstrated a significant reduction (*P* < 0.05) in parasite burden caused by the RHΔ*ompdc*Δ*uprt* mutant*.* Next, the histological sections of liver, spleen, and lung tissues were subjected to HE staining for pathological examination. As shown in Fig. 3f, few changes were observed in tissue sections from RHΔ*ompdc*Δ*uprt*-injected mice compared with naïve mice, while in wild type infected mice, large amounts of necrotic cells and absent lymphoid follicles were appeared in spleen tissue, obvious cellular separation was displayed in liver, and notably thicker alveolar walls were observed in lung tissues.

### Robust humoral and cellular immune responses were elicited by vaccination with the RHΔ*ompdc*Δ*uprt* mutant in mice

**Fig. 4:**
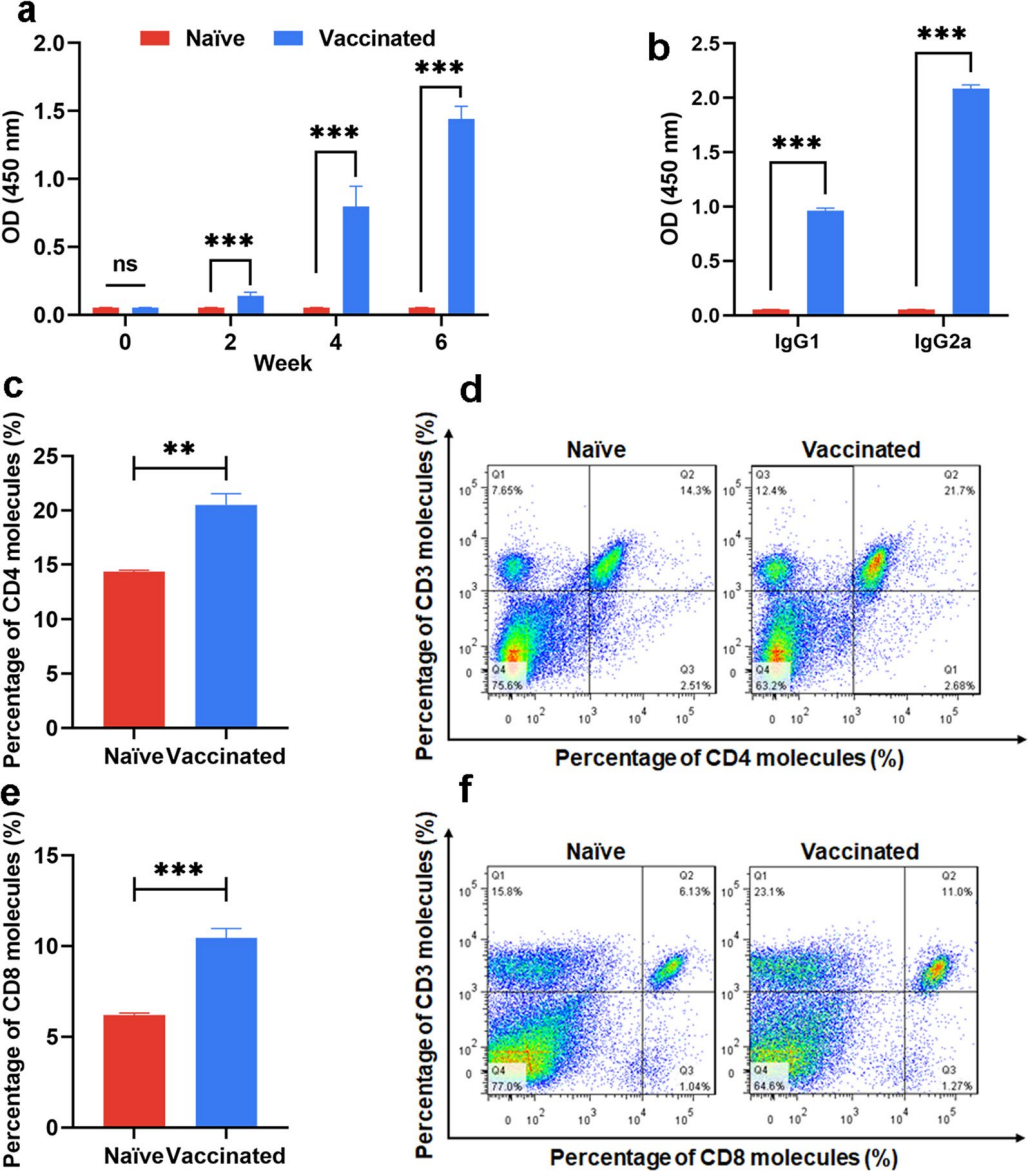
Immunization with the RHΔ*ku80*Δ*ompdc*Δ*uprt* mutant vaccine induced specific humoral and cellular responses. **a** Determination of IgG antibodies in the sera of BALB/c mice at 0, 2, 4, and 6 weeks. **b** Detection of antibody subtypes (IgG1 and IgG2a) in the sera of immunized mice 2 weeks after the last immunization. **c**–**f** Percentages of CD4^+^ T cells and CD8^+^ T cells subsets in immunized BALB/c mice. The results are shown as the means ± *SD* from three independent experiments. *P < 0.05, **P < 0.01 ***P < 0.001 by Student’s *t* test, ns not significant. *SD* standard deviation

Significantly high levels of anti-*T. gondii* IgG were detected in vaccinated mice, and the IgG titer was increased remarkably (*P* < 0.05) after every vaccination (Fig. 4b), indicating that a robust humoral response was induced. The levels of IgG subclasses (IgG1 and IgG2a) were tested to characterize the immune response type. Both the levels of IgG1 and IgG2a were significantly higher (*P* < 0.05) in RHΔ*ompdc*Δ*uprt*-vaccinated mice than in control mice (Fig. 4c). In addition, the level of IgG2a was notably higher than that of IgG1, indicating that vaccination with RHΔ*ompdc*Δ*uprt* in mice elicited a Th1/Th2 mixed and Th1-biased immune response.

**Fig. 5:**
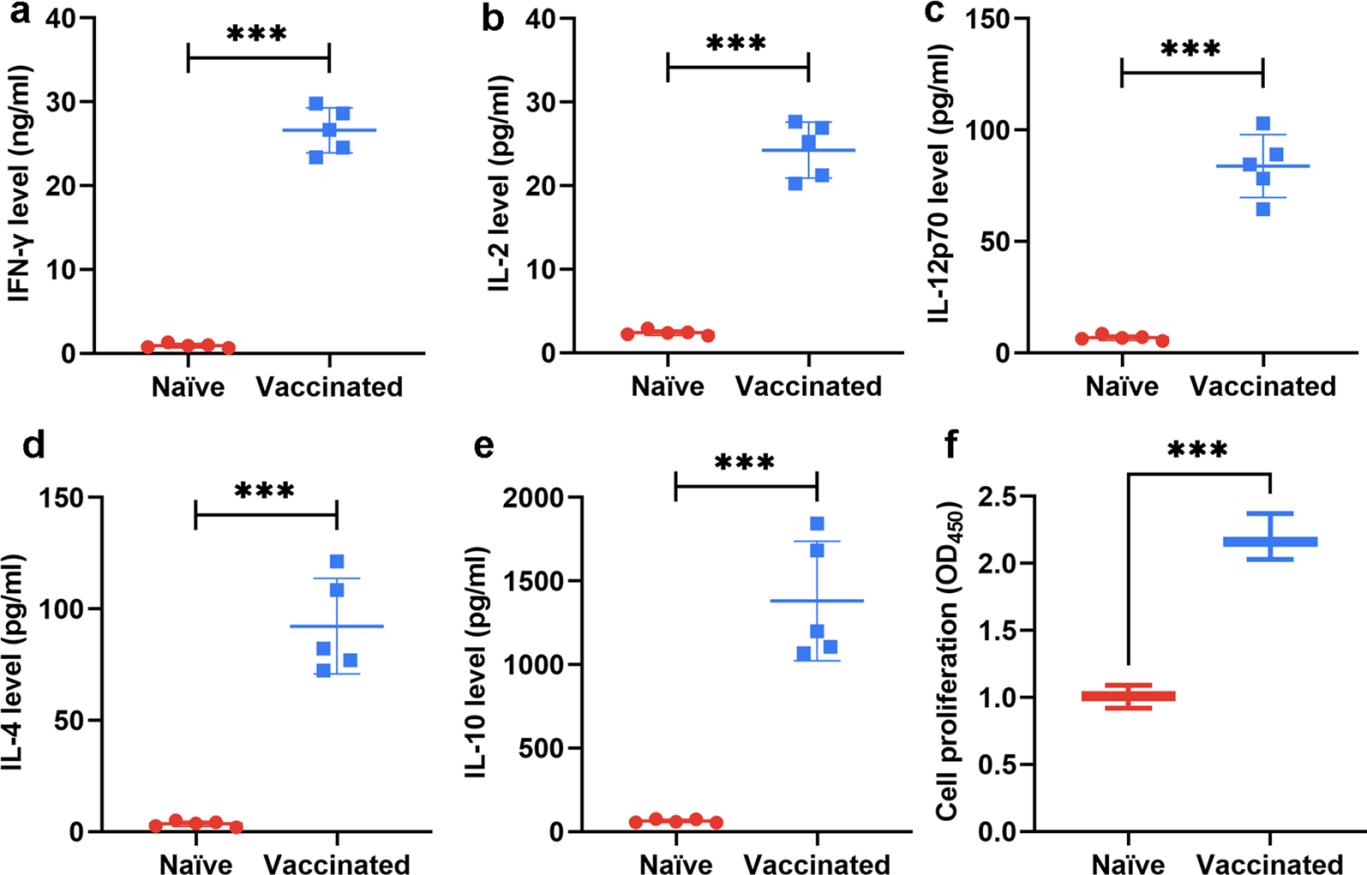
Pro-Inflammatory cytokines were elicited by immunization with RHΔ*ku80*Δ*ompdc*Δ*uprt*. Splenocytes collected from the immunized and non-immunized (6 weeks after immunization) mice were co-incubated with STAg (10 µg/ml). The levels of Th1 [IFN-γ (**a**), IL-2 (**b**), and IL-12 (**c**)] and Th2 [IL-4 (**d**) and IL-10 (**e**)] in the culture supernatants were measured by flow cytometry. (**f**) The proliferative responses of splenocytes were measured by a CCK8 kit. The results are presented as the means ± *SD*. (*n* = 5, ***P < 0.001 by Student’s *t* test). *STAg* soluble *Toxoplasma gondii* antigens, *CCK* Cell counting kit, *SD* standard deviation

After immunization with the RHΔ*ompdc*Δ*uprt* mutant, a significant increase (*P* < 0.05) in the percentage of CD3^+^CD4^+^ T cells by 2 weeks post vaccination was observed compared with that of the controls (Fig. 4d, e). Additionally, the percentage of CD3^+^CD8^+^ T cells in vaccinated mice was increased to a much higher degree (*P* < 0.05) (Fig. 4f, g). In parallel, the rest of splenocytes from vaccinated and unvaccinated mice were cultured in vitro for further stimulation with STAg. The cytokine levels in the splenocyte supernatant were then detected by flow cytometry. Consistent with the levels of IgG1 and IgG2a, the proinflammatory cytokine levels, including Th1-type cytokines (IL-2, IL-12, IFN-γ) and Th2-type cytokines (IL-10, IL-4) of the immunized mice were notably higher (IL-2: 24.27 ± 3.35 pg/ml; IL-12: 83.83 ± 14.15 pg/ml; IFN-γ: 26.61 ± 2.68 ng/ml; IL-10: 1380 ± 357.7 pg/ml; IL-4: 92.22 ± 21.46 pg/ml) than those of the control mice (*P* < 0.05) (Fig. 5a–e). The results also showed a quick and robust proliferation (*P* < 0.05) of splenocytes once stimulated by the STAg (Fig. 5f), indicating the efficient cellular immune response induced by the immunization of the mutant. Of note, IL-12 and IFN-γ which are the key factors in cellular immune clearance of tachyzoites were also found significantly increased (*P* < 0.05) in sera samples of immunized mice and lasted for nearly a week post vaccination (Additional file 1: Fig. S4).

### RHΔ*ompdc*Δ*uprt* immunization confers protection against infection with various types of *T. gondii* tachyzoites in mice

**Fig. 6:**
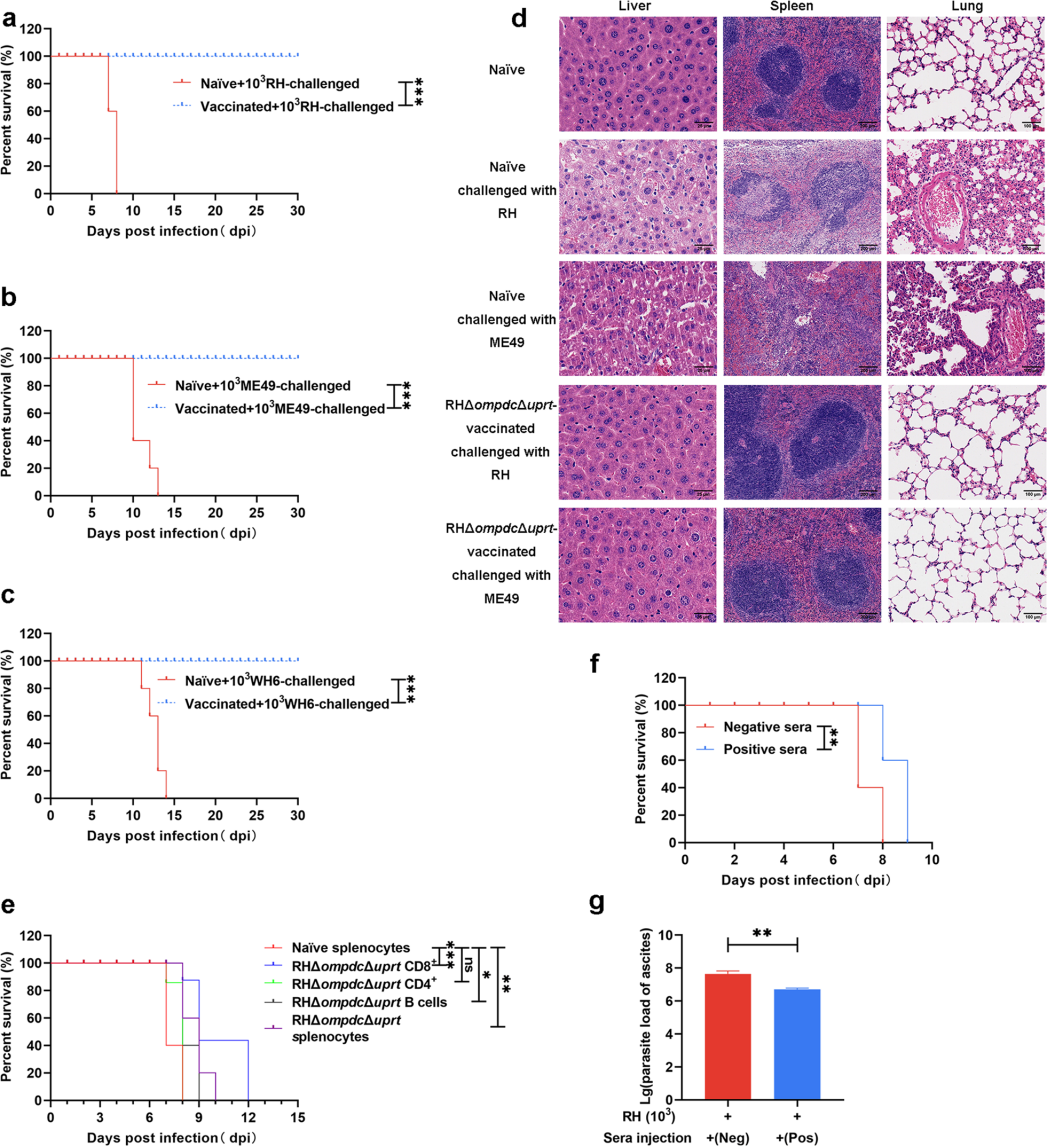
Protective immunity induced by the RHΔ*ku80*Δ*ompdc*Δ*uprt* vaccine against *T. gondii* challenge infection in mice. **a**–**c** Survival curves of naïve or RHΔ*ku80*Δ*ompdc*Δ*uprt* immunized mice infected with RHΔ*ku80*, ME49 and WH6. Two weeks after the last immunization, mice were intraperitoneally (i.p.) challenged with 1 × 10^3^ T*. gondii* tachyzoites of RHΔ*ku80*, ME49 and WH6 strains (5 mice/strain), and monitored for 30 days. **d** Organ damage in vaccinated mice and naïve mice after challenge with the RHΔ*ku80* and ME49 strains. **e** Two weeks after the last immunization, whole splenocytes, CD4^+^, CD8^+^ and CD19^+^ splenocytes were harvested, and 1 × 10^7^ total immune splenocytes, 2 × 10^6^ CD8^+^ T cells, 2 × 10^6^ CD4^+^ T cells, 2 × 10^6^ CD19^+^ B cells, or 1 × 10^7^ total naïve splenocytes were transferred to naïve recipient mice. Twenty-four hours after transfer mice mice were challenged with 1 × 10^3^ T*. gondii* RHΔ*ku80* i.p. and monitored for survival. **f** From day 0 to day 4 after infection, mice were treated with sera from RHΔ*ku80*Δ*ompdc*Δ*uprt*-vaccinated and naïve mice. **g** Parasite load detection after one day of sera treatment. *P < 0.05, **P < 0.01, *** *P* < 0.001 by log-rank (Mantel–Cox) test, ns not significant

Now that the above results demonstrated that the strong immune responses were successfully stimulated, the protective efficacy provoked by the RHΔ*ompdc*Δ*uprt* mutant was evaluated. Two weeks after the third vaccination, naïve and vaccinated mice were challenged with lethal doses of type I RHΔ*ku80* strains. All naïve mice died within 10 days, while 100% of the vaccinated mice completely survived (Fig. 6a). In addition, when challenged with type II ME49 tachyzoites or Chinese locally isolated strain WH6, RHΔ*ompdc*Δ*uprt* mutant-vaccinated mice presented 100% survival, while naïve mice all died within 13 or 14 days post infection (Fig. 6b, c). Subsequently, tissues including liver, spleen, and lung were collected for pathological examination after 6 days (RHΔ*ku80*) or 12 days (ME49) of challenged with tachyzoites. Obvious changes were observed in unvaccinated mice, such as liver cell necrosis, spleen body destruction, and lung congestion as described above, while the tissue sections of vaccinated mice showed limited changes (Fig. 6d).

### The immune protection induced by RHΔ*ompdc*Δ*uprt* vaccination could be adoptively transferred against acute infection in mice

Mice that received naïve splenocytes or RHΔ*ompdc*Δ*uprt*-vaccinated CD4^+^ T cells succumbed to death by 9 dpi (Fig. 6e), in contrast, the passive immunization of purified CD19^+^ B cells, CD8^+^ T cells, or total splenocytes from RHΔ*ompdc*Δ*uprt*-vaccinated mice survived a significantly longer (*P* < 0.05) time than naïve mice (Fig. 6e). The data showed a relative but significantly longer (*P* < 0.05) survival rate in passively immunized mice than in control mice (Fig. 6f). Consistent with this result, a significantly lower parasite load was observed in passively immunized mice (Fig. 6g).

### Vaccination with the RHΔ*ompdc*Δ*uprt* mutant was determined to be safe in cats and to induce a robust immune response

**Fig. 7:**
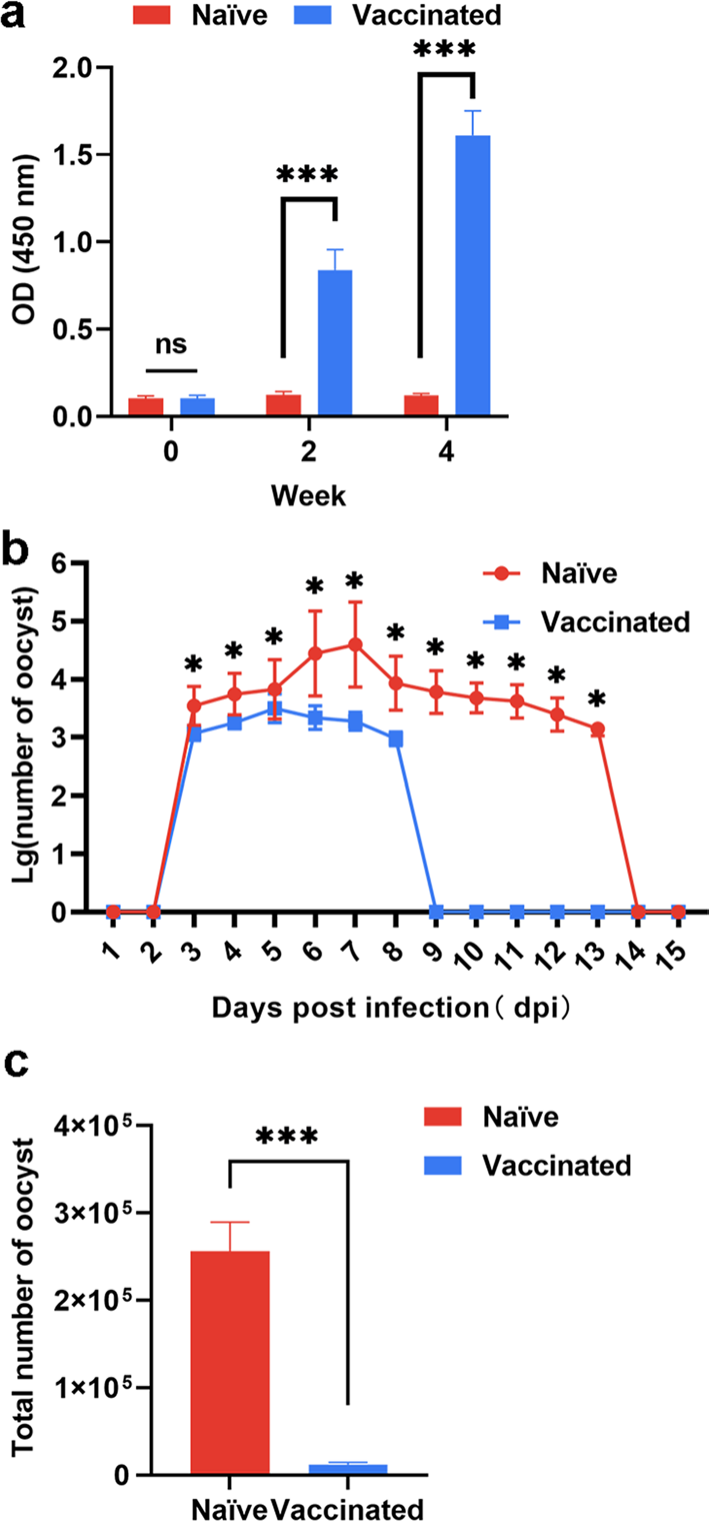
Protective immunity induced by the RHΔ*ku80*Δ*ompdc*Δ*uprt* vaccine against *T. gondii* challenge infection in cats. **a** Determination of IgG antibodies in the sera of cats at 0, 2 and 4 weeks. **b** Daily oocyst emissions of naïve or RHΔ*ku80*Δ*ompdc*Δ*uprt* immunized cats orally administered 200 cysts of ME49. **c** Total oocyst emissions of naïve or RHΔ*ku80*Δ*ompdc*Δ*uprt* immunized cats orally administered 200 cysts of ME49. (*n* = 6, *P < 0.05, ***P < 0.001 by Student’s *t* test, ns not significant.)

As showed in Table 2S, no oocysts were found in any of the RHΔ*ompdc*Δ*uprt*-injected cats. Then, the immune response in cats was further evaluated. After vaccination for 2 doses, sera obtained from cats were used to determine the specific antibody response. Significantly high levels of anti-*T. gondii* IgG were detected in vaccinated cats, and the IgG titer was increased remarkably (*P* < 0.05) after every vaccination (Fig. 7a), indicating that a robust humoral response was induced by the mutant.

### RHΔ*ompdc*Δ*uprt*-vaccinated cats notably reduced the oocyst shed number and period

To evaluate the possibility of applying the vaccine in cats, we performed a challenge and tested the expulsion of oocysts. Oocysts were detectable at 3 dpi for all-infected cats, while RHΔ*ompdc*Δ*uprt*-vaccinated cats notably reduced (*P* < 0.05) the shedding period (6 days) compared with that of naïve cats (11 days) as shown in Fig. 7b. Furthermore, the results showed a remarkable decrease (*P* < 0.05) in the total number of oocysts (95.3%) in vaccinated cats compared with naïve cats (Fig. 7c).

## Discussion

Control of *T. gondii* is a major challenge since the parasite can cross the blood–brain barrier to develop a persistent infection where most chemicals are inaccessible [31]. Currently, the combination of pyrimethamine and sulfadiazine is commonly used clinically for the treatment of acute toxoplasmosis [32]. However, these drugs have limited effects on *T. gondii* cysts. It has become a popular theory that preventative strategies such as vaccination are a more effective way to provide protection against *T. gondii*. Attempts to develop vaccines over the last 60 years have acquired a few successful experimental candidates, among which live attenuated vaccines are the most promising ones. Live attenuated vaccines are non-disease cause mutants created by weakening or altering the pathogen. In this study, we applied the double gene knockout strain RHΔ*ompdc*Δ*uprt* as a live attenuated vaccine and explored the induction of protective immunity against *T. gondii* challenge.

Undoubtedly, safety and effectiveness are the primary factors for a vaccine. Currently, there is only one vaccine called *Toxovax* on the market specifically derived from the S48 strain to reduce fetal abortion in sheep. However, this vaccine is not safe enough to use in humans due to the possibility that the strain may revert the ability to form cysts as the data show that it could cause acute infection and lead to death in murine models [33]. Recently, several studies with emphasis on gene modified mutants proved that robust protective immune responses had been elicited by these vaccines, such as RHΔ*gra17*Δ*ntp1* [17] and ME49Δ*ldh* [34] and the uracil auxotroph mutants* (*Δ*cpsII* and Δ*ompdc)* [20, 23]*.* With defective de novo UMP biosynthesis activity, uracil auxotroph mutants lose the ability to propagate in vivo and induce long-term protection against acute and chronic *T. gondii* infection. However, we cannot ignore the fact that their protection is not always 100%. As reported by Peng [24], the Δ*ompdc* strain supplied without uracil could still replicate relatively slow but never stop way in vitro, indicating that the salvage pathway may play a compensatory role. Although they concluded that in *ompdc* disrupted strains, the potential salvage of host cell uracil and nucleosides is not sufficient to support a significant rate of parasite replication in vitro [23], safety cannot be guaranteed when this live attenuated vaccine is used in meat producing animals or cats. Thus, according to the UMP synthesis and salvage pathways, we specifically deleted the *ompdc* and *uprt* through a CRISPR/Cas9 system to completely block the production or intake of UMP. Consistent with the predicted model, the RHΔ*ompdc*Δ*uprt* strain lost viability in vitro, as confirmed by parasite replication and plaque assays. Furthermore, no deaths were observed when the RHΔ*ompdc*Δ*uprt* strains were subjected to BALB/c or BALB/c-nu mice, demonstrating that this strain is safe in immunodeficient individuals. As documented and evaluated here, the tachyzoites could be found in ascitic fluids as early as 1 dpi in acute infection mice and parasite loads in tissues such as liver and lung increased notably [35]. However, we found a rather small number of parasites in ascitic fluids and tissues, which means that the RHΔ*ompdc*Δ*uprt* strain is avirulent to mice. Additional visual evidence is that limited pathological changes or tachyzoites were found in histological sections from RHΔ*ompdc*Δ*uprt* strain injected mice. To our knowledge, once infected by *T. gondii*, mouse tissues display obvious changes, such as clear cellular separation in the liver indicting hepatocellular dysfunction, thicker alveolar walls in the lung representing interstitial pneumonia, and plenty of necrotic cells in the spleen with absent lymphoid follicles [36]. Thus, this avirulent RHΔ*ompdc*Δ*uprt* strain is nearly harmless to mice. In addition, the daily analysis of cat feces after vaccination with the RHΔ*ompdc*Δ*uprt* strain revealed no oocysts, indicating that this attenuated mutant also lost the ability of sexual reproduction compared with previous results [37]. Consequently, we conclude that the attenuated strain is unable to reproduce in mouse and cat models with limited harm or side effects.

Effectiveness is a more crucial factor for an ideal vaccine. We noticed that the type of attenuated *T. gondii* strain for vaccine development is also of great importance. As reported by Xia et al. [34], the ME49Δ*ldh* mutant induced long-term protection against type 2 and 3 strains, while short-term protection against the type 1 RH strain. Therefore, we chose the RH strain to construct the live attenuated vaccine. Consistently, this defective vaccine contributed 100% protection against the RHΔ*ku80*, ME49 and WH6 strains through a humoral and cellular mixed immune response in a murine model. In consideration of the data, we gathered above that RHΔ*ompdc*Δ*uprt* would be cleared within 1 week, we proceeded with a three-dose inoculation procedure rather than a single inoculation procedure, as reported by others [14, 22, 24, 34] to achieve a higher and longer immune response. As expected, high levels of specific anti-*T. gondii* IgG antibodies were gradually induced by the RHΔ*ompdc*Δ*uprt* strain, particularly both IgG2a and IgG1 were significantly increased indicating a mixed Th1/Th2 immune response which is consistent with other live attenuated *T. gondii* vaccines [17, 38, 39]. These specific antibodies play a protective role by neutralizing the attachment of *T. gondii*. [40–43]. In addition, the level of IgG2a was significantly higher than that of IgG1, suggesting that the immunized mice successfully induced a significant Th1-type humoral immune response. This is consistent with previous reports that IgG2a is more efficient than IgG1 in clearing *T. gondii* tachyzoites [44]. In addition, we demonstrated that the adoptive transfer of sera from RHΔ*ompdc*Δ*uprt* immunized mice to naïve recipients provided short-term protection against virulent challenge. The protection of sera is most likely provided by the IFN-γ and IL-12p70 cytokines since we observed that they were significantly induced by the injection of the RHΔ*ompdc*Δ*uprt* strain and sustained for a quite long period, but on the other hand, the eventual death of mice was probably due to the depletion of cytokines succumbing to virulent challenge.

Understanding the mechanism of immunity is essential for the development of vaccines. Since immunity has been correlated with a Th1 biased inflammatory response to *T. gondii*, most vaccines attempt to achieve a higher Th1 response. Evidence from other studies particularly supports that CD8^+^ T cells and IFN-γ are absolutely essential to combat *T. gondii* infection. Our analysis indicated that both CD4^+^ and CD8^+^ T cell subsets were recruited by RHΔ*ompdc*Δ*uprt.* Despite the fact that both CD4^+^ and CD8^+^ T cells correspond to the production of IFN-γ, infected mice developed partial protection against RHΔ*ku80* challenge provided by passive immunization of purified CD8^+^ T cells instead of CD4^+^ T cells. Interestingly, we also established that adoptive transfer of purified B cells to naïve recipients provided protection against virulent challenge. However, the protective effective of cells, as well as sera, from RHΔ*ku80*-infected mice remained unclear since these passive immunizations were difficult to carried out due to the persistent presence of parasites in these cells that could result in *T. gondii* infection. Collectively with the results of the humoral protective immune response above, we assume that protective immunity would be enhanced by memory B cells through a more efficient antigen processing and presentation producer via multi-point inoculation. Therefore, a CD8^+^ cells dominant and B cells required immunity was elicited in response to vaccination with RHΔ*ompdc*Δ*uprt* for the protection of *T. gondii.*

IFN-γ can induce various intracellular mechanisms to kill parasites or inhibit their replication and proliferation [45–47]. We observed that the expression levels of IFN-γ in the culture supernatant of splenocytes and sera from the vaccinated mice were remarkably higher than those of the unvaccinated mice, which in turn provided effective protection against a high dose virulent challenge. Correspondingly, we found that mice vaccinated with RHΔ*ompdc*Δ*uprt* exhibited 100% protection against type I, type II, or Chinese isolated strains, in accordance with the protective effects of the Δgra7Δ*npt1* and Δ*cpsII* strains, while those immunized with ME49Δ*ldh* or ME49Δ*cdpk3* [48] only provided short-term protection against the type I RH strain. In addition, we also revealed that RHΔ*ompdc*Δ*uprt* can induce and sustain significant IL-12p70 production, indicating that the innate immune response is also activated. IL-12p70 is mainly produced by B cells and neutrophils, as reported by Gigley [39]. Infection with live attenuated tachyzoites recruits neutrophils early and acts as an innate effector cell, resulting in the early secretion of IL-12 to destroy intracellular parasites by autophagy. Our data indicate a rapid increase in IL-12p70 and a daily decrease in response to vaccination with RHΔ*ompdc*Δ*uprt,* while IFN-γ presents a gradually increasing pattern with a peak production level at 4 dpi. However, other studies have shown that excessive levels of Th1 inflammatory factors may also cause pathological damage to mice or even lead to the death of mice [49, 50]. At this time, IL-10 and IL-4 are needed to regulate the inflammatory response during *T. gondii* infection [51–53]. In accordance with this theory, we discovered that Th2-type cytokines (IL-4 and IL-10) were notably increased in response to RHΔ*ompdc*Δ*uprt* infection to balance the high production of Th1 cytokines. As the results, immunized mice developed immune profiles capable of clearing high doses of type I and type II *T. gondii* tachyzoites challenge with limited pathological change.

It is well known that as the definitive host, cats infected with *T. gondii* can excrete a large number of infective oocysts with feces lasting for about two weeks, while there are few studies on anti-*T. gondii* vaccines in cats. Given the crucial role of cats in the transmission of *T. gondii*, there is an urgent need to develop vaccines for cats. The mutant of the *T. gondii* bradyzoite (T-263) was the first report of a feline *T. gondii* vaccine [54]. By oral administration of T-263 bradyzoites obtained from brain cysts, 84% of immunized cats excreted no oocysts [54], while immunization with T-263 tachyzoites did not completely induce protective immunity against oocyst shedding [55]. We should not ignore that vaccination with brayzoites from brain cysts is impractical in clinical use in consideration of production and cost. Later, some studies have shown that rhoptry protein vaccines could induce a 67% preventable fraction of oocysts in cats [56]. In the present study, we found that cats immunized with this live attenuated strain developed high antibody titers and showed a 95.3% reduction in oocyst shedding after challenge. Of note, all cats were successfully immunized with a significantly shorter period of oocyst shedding indicating effective protection against toxoplasmosis in cats.

Although RHΔ*ompdc*Δ*uprt* strains as attenuated live vaccine showed a protective effect against feline toxoplasmosis, they failed to reach the 100% blocking level of oocyst, which may be related to the immune dose level, immune frequency and immune interval. In the future, we expect to further enhance immune protection by optimizing the immune program.

## Conclusions

Our study shows that the attenuated *ompdc*-*uprt* double knockout strains from the RH strain of *T. gondii* are safe and avirulent, can protect mice from challenge with high doses of RHΔ*ku80*, ME49 and WH6 strain tachyzoites and can allow the cat to reduce the excretion of oocysts. These data suggest that the RHΔ*ompdc*Δ*uprt* mutant has the potential to be used as a candidate for a live attenuated vaccine. Although this mutant strain has great potential as a vaccine in mice, we also need to further study whether this vaccine has similar efficacy in other animals.

## Supplementary Information

**Additional file 1: Table S1.** The primers used in this study. **Table S2.** This table shows the oocysts in the feces of cats from 1 to 10 days after being infected with RHΔ*ompdc*Δ*uprt* strain. **Figure S1.** The diagram of knocking out. (a) Schematic illustration of knocking out *uprt* by homologous gene replacement in RHΔ*ku80*Δ*hxgprt* strain. (b) Schematic showing deletion of *hxgprt* gene by insertion of a *uprt*5’utr-3’utr into *hxgprt* gene. (c) Diagram illustrating the deletion of *ompdc* in RHΔ*uprt*Δ*hxgprt* to make the double mutant RHΔ*uprt*Δ*ompdc*::HXGPRT by CRISPR/Cas9-mediated homologous gene replacement. **Figure S2.** Intracellular proliferation of OMPDC-UPRT deletion mutant under the fluorescence microscope. (a) 24 hours of intracellular proliferation. (b) 48 hours of intracellular proliferation. **Figure S3.** Invasion and attachment assay for RHΔ*ku80* and RHΔ*ompdc*Δ*uprt* strains. (a) The invasion and attachment of *T. gondii* RHΔ*ku80* and RHΔ*ompdc*Δ*uprt* were evaluated by indirect immunofluorescence assay. (b) The analysis of the invasion and attachment. **Figure S4.** The levels of cytokines in the serum of mice injected with RHΔ*ompdc*Δ*uprt*. The levels of IFN-γ (a) and IL-12 (b) were measured by flow cytometer.

## Data Availability

The original data that supports the conclusions of this article are presented in the article or in the supplementary information.

## Abbreviations

OMPDC: Orotidine—5'—monophosphate decarboxylase
UPRT: Uracil phosphoribosyltransferase
UMP: Uridine monophosphate
HFFs: Human foreskin fibroblasts
DMEM: Dulbecco’s Modified Eagle medium
i.p.: Intraperitoneally
FBS: Fetal bovine serum
PBS: Phosphate buffered saline
IFA: Immunofluorescence assay
ELISA: Enzyme-linked immunosorbent assay
TMB: 3,3′,5,5′-Tetramethylbenzidine
CBA: Cytometric bead array
CCK-8: Cell counting kit 8
PV: Parasitophorous vacuole
