# Supplementary material for: A live attenuated RHΔompdcΔuprt mutant of Toxoplasma gondii induces strong protective immunity against toxoplasmosis in mice and cats
Source: Infect Dis Poverty. 2023 Jun 15;12:60. doi: 10.1186/s40249-023-01109-9 (PMC10266960; doi:10.1186/s40249-023-01109-9)
Supplement: Supplementary file 1 — Additional file 1: Table S1. The primers used in this study. Table S2. This table shows the oocysts in the feces of cats from 1 to 10 days after being infected with RHΔompdcΔuprt strain. Figure S1. The diagram of knocking out. (a) Schematic illustration of knocking out uprt by homologous gene replacement in RHΔku80Δhxgprt strain. (b) Schematic showing deletion of hxgprt gene by insertion of a uprt5’utr-3’utr into hxgprt gene. (c) Diagram illustrating the deletion of ompdc in RHΔuprtΔhxgprt to make the double mutant RHΔuprtΔompdc::HXGPRT by CRISPR/Cas9-mediated homologous gene replacement. Figure S2. Intracellular proliferation of OMPDC-UPRT deletion mutant under the fluorescence microscope. (a) 24 hours of intracellular proliferation. (b) 48 hours of intracellular proliferation. Figure S3. Invasion and attachment assay for RHΔku80 and RHΔompdcΔuprt strains. (a) The invasion and attachment of T. gondii RHΔku80 and RHΔompdcΔuprt were evaluated by indirect immunofluorescence assay. (b) The analysis of the invasion and attachment. Figure S4. The levels of cytokines in the serum of mice injected with RHΔompdcΔuprt. The levels of IFN-γ (a) and IL-12 (b) were measured by flow cytometer. [file 40249_2023_1109_MOESM1_ESM.pdf]

Supplementary information

Supplementary Table1. The primers used in this study.

Primer	Sequence	Use
sgRNA-HXGPRT-F	5'-GATCGCTCCAACAGCTTGAAG TTTTAGAGCTAGAAATAGC-3'	To construct the HXGPRT specific CRISPR plasmid
sgRNA-OMPDC-F	5'-GAAGACTCTCCATCCACGACG TTTTAGAGCTAGAAATAGC-3'	To construct the OMPDC specific CRISPR plasmid
sgRNA-R	5'–AACTTGACATCCCCATTTAC–3'	To construct the HXGPRT and OMPDC specific CRISPR plasmid
HX-UPRT-F	5'-TATTCCTTTTTTCGTCGGACCTT TCCACAGGGCTTCTAAAGAT CAGCACGAAACCTTGCA-3'	Amplification of 5′-homology of UPRT
HX-UPRT-R	5'-TCCGCGATTCCGTCAGCGGTCT GTCAAAAAAACTAGAGACG TGGATCCCCCTCCACCGCG-3'	Amplification of 3′-homology of UPRT
UPRT-5'utr-F	5'-GAGCAACTTACAAGGACAAGG-3'	To construct the homology of UPRT3'utr-5'utr
UPRT-3'utr5'utr-R	5'-CCGTCAGCGGTCTTTTAGAAGC CCTGTGGA-3'	To construct the homology of UPRT3'utr-5'utr
UPRT-5'utr3'utr F	5'-CAGGGCTTCTAAAAGACCGCTG ACGGAATC-3'	To construct the homology of UPRT3'utr-5'utr
UPRT-3'utr R	5'-TCTTCACATTGCTTCAACAA-3'	To construct the homology of UPRT3'utr-5'utr
HX-OMPDC-F	5'-GACATTTTTCCGTGACAATTCCA AAGAACTGTCAAATAAGGAT CAGCACGAAACCTTGCA-3'	Amplification of 5′-homology of OMPDC
HX-OMPDC-R	5'-ATGTAGACCAGAGTCACGATAGT GTTTCGCTTTCAGCACGGTG GATCCCCCTCCACCGCG-3'	Amplification of 3′-homology of OMPDC
UPRT-F1	5'-GCGGTAAGACGAGGGCCATATC-3'	PCR2 of Δ*ku80*Δ*hxgprt*

		and Δ*uprt*::HXGPRT
UPRT-R1	5’-GAAGATTATTCCGCACCTTAC-3’	PCR2 of Δ*ku80*Δ*hxgprt* and Δ*uprt*::HXGPRT
UPRT-F2	5’-GTTGTGTCTGTGGAGGAAG-3’	PCR2 of Δ*uprt*::HXGPRT and Δ*uprt*Δ*hxgprt*
UPRT-R2	5’-ATGTCGTCGCAGTAGTGA-3’	PCR2 of Δ*uprt*::HXGPRT and Δ*uprt*Δ*hxgprt*
529bp-F	5’-CGCTGCAGGGAGGAAGACGA AAGTTG-3’	PCR3 of Δ*uprt*::HXGPRT and Δ*uprt*Δ*hxgprt*
529bp-R	5’-CGCTGCAGACACAGTGCATCT GGATT-3’	PCR3 of Δ*uprt*::HXGPRT and Δ*uprt*Δ*hxgprt*
HXGPRT-F	5’-GAGCACTATGTCCGCCTGAA-3’	PCR1 of Δ*uprt*::HXGPRT, Δ*uprt*Δ*hxgprt* and Δ*ompdc*Δ*uprt*::HXGPRT
HXGPRT-R	5’-TGTACACTTAGGTGTCGCGG-3’	PCR1 of Δ*uprt*::HXGPRT, Δ*uprt*Δ*hxgprt* and Δ*ompdc*Δ*uprt*::HXGPRT
OMPDC-F1	5’-GATAAGTCGCACGCCAGAGGAG-3’	PCR2 of Δ*uprt*Δ*hxgprt* and Δ*ompdc*Δ*uprt*::HXGPRT
OMPDC-R1	5’-TCGCACGCATCTCAGATTGTCA-3’	PCR2 of Δ*uprt*Δ*hxgprt* and Δ*ompdc*Δ*uprt*::HXGPRT
OMPDC-F2	5’-GGTTGTCCAGGCTGCTGTGAAT-3’	PCR3 of Δ*uprt*Δ*hxgprt* and Δ*ompdc*Δ*uprt*::HXGPRT
OMPDC-R2	5’-CTATCCGTGGTGACTGCGAAGG-3’	PCR3 of Δ*uprt*Δ*hxgprt* and Δ*ompdc*Δ*uprt*::HXGPRT
RT-OMPDC-F	5’-CGCATCATCAGGGAAACTCTTC-3’	qRT-PCR of Δ*ompdc*Δ*uprt*::HXGPRT
RT-OMPDC-R	5’-TAGGAACATCGTCGGGAATCAG-3’	qRT-PCR of Δ*ompdc*Δ*uprt*::HXGPRT
RT-UPRT-F	5’-TTTCCCAATGTGGTGCTC-3’	qRT-PCR of Δ*ompdc*Δ*uprt*::HXGPRT
RT-UPRT-R	5’-GCTTCTTCGATGAGGAGG-3’	qRT-PCR of Δ*ompdc*Δ*uprt*::HXGPRT

RT-GAPDH-F	5’-ATTTTGCTTGGGATTCGAGGA-3’	qRT-PCR of GAPDH
RT-GAPDH-R	5’-TGCAGGGTAACGATCAAAAAATG-3’	qRT-PCR of GAPDH
Q529bp-F	5’-CACAGAAGGGACAGAAGT-3’	529bp-based qPCR
Q529bp-R	5’-TCGCCTTCATCTACAGTC-3’	529bp-based qPCR

Supplementary Table 2 (Table 2S). This table shows the oocysts in the feces of cats from 1 to 10 days after being infected with RHΔ*ompdc*Δ*uprt* strain.

**Days post infection**	**1**	**2**	**3**	**4**	**5**	**6**	**7**	**8**	**9**	**10**
**Blank**	**-**	**-**	**-**	**-**	**-**	**-**	**-**	**-**	**-**	**-**
**RHΔ*ompdc*Δ*uprt***	**-**	**-**	**-**	**-**	**-**	**-**	**-**	**-**	**-**	**-**

-： no oocysts.

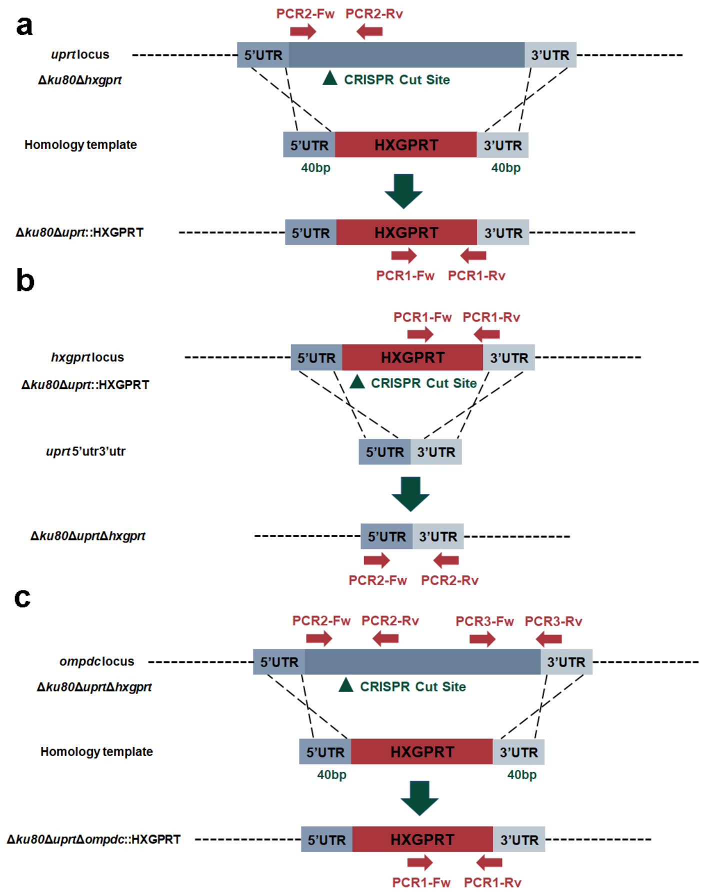

Supplementary Figure 1 (Fig.1S). The diagram of knocking out. **(a)** Schematic illustration of knocking out *uprt* by homologous gene replacement in RHΔ*ku80*Δ*hxgprt* strain. **(b)** Schematic showing deletion of *hxgprt* gene by insertion of a *uprt*5'utr-3'utr into *hxgprt* gene. **(c)** Diagram illustrating the deletion of *ompdc* in RHΔ*uprt*Δ*hxgprt* to make the double mutant RHΔ*uprt*Δ*ompdc*::HXGPRT by CRISPR/Cas9-mediated homologous gene replacement.

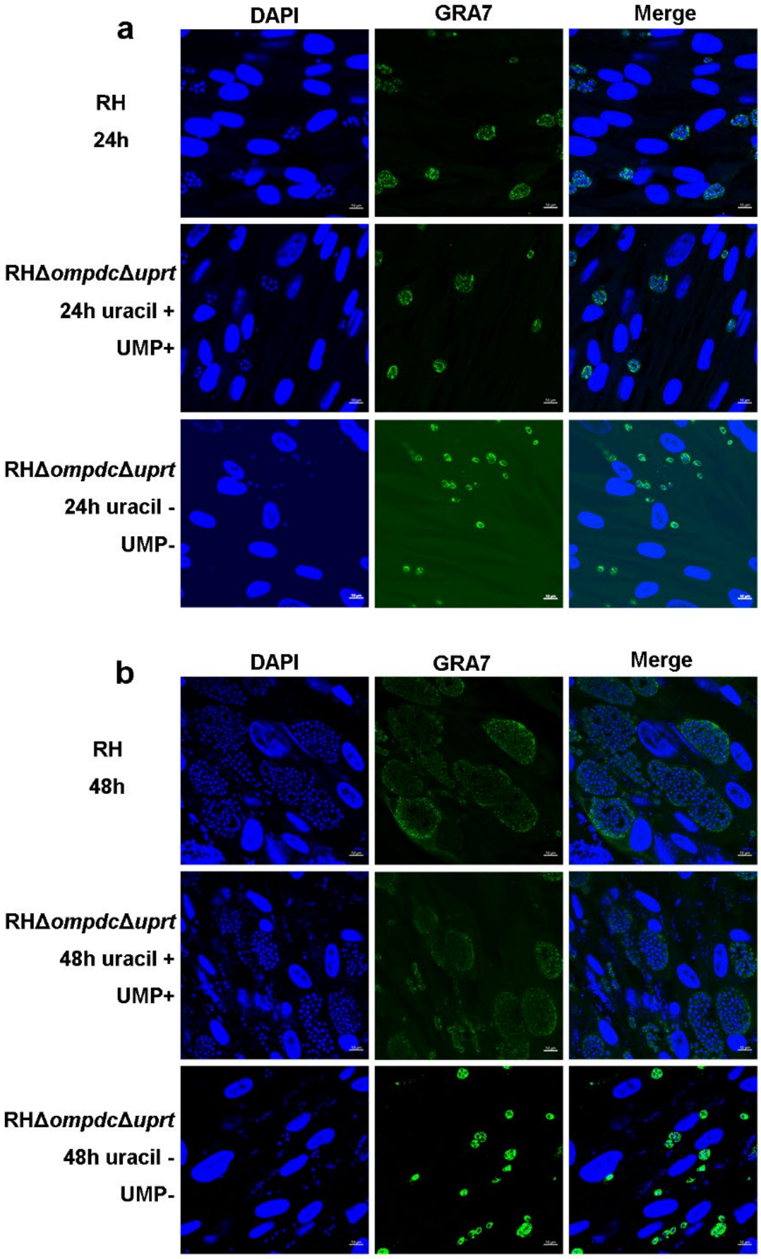

Supplementary Figure 2 (Fig.2S). Intracellular proliferation of OMPDC-UPRT deletion mutant under the fluorescence microscope. **(a)** 24 hours of intracellular proliferation. **(b)** 48 hours of intracellular proliferation.

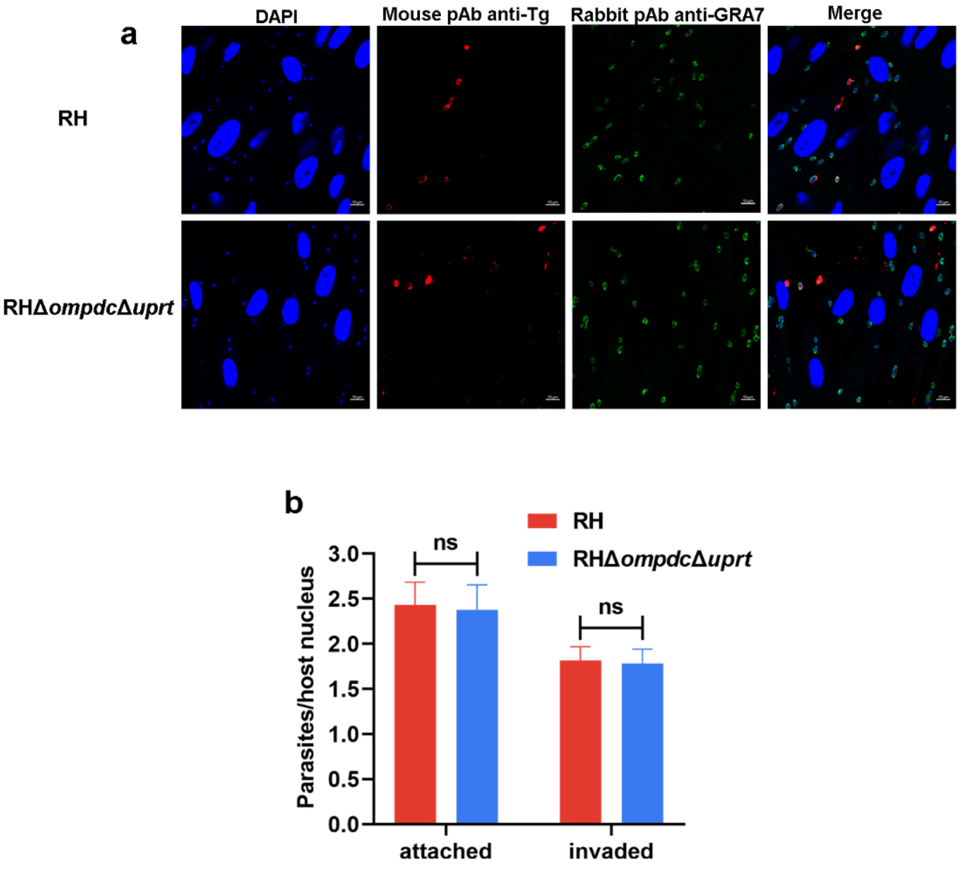

Supplementary Figure3 (Fig.3S). Invasion and attachment assay for RHΔ*ku80* and RHΔ*ompdc*Δ*uprt* strains. **(a)**The invasion and attachment of *T.gondii* RHΔ*ku80* and RHΔ*ompdc*Δ*uprt* were evaluated by indirect immunofluorescence assay (IFA). **(b)** the analysis of the invasion and attachment. ns not significant.

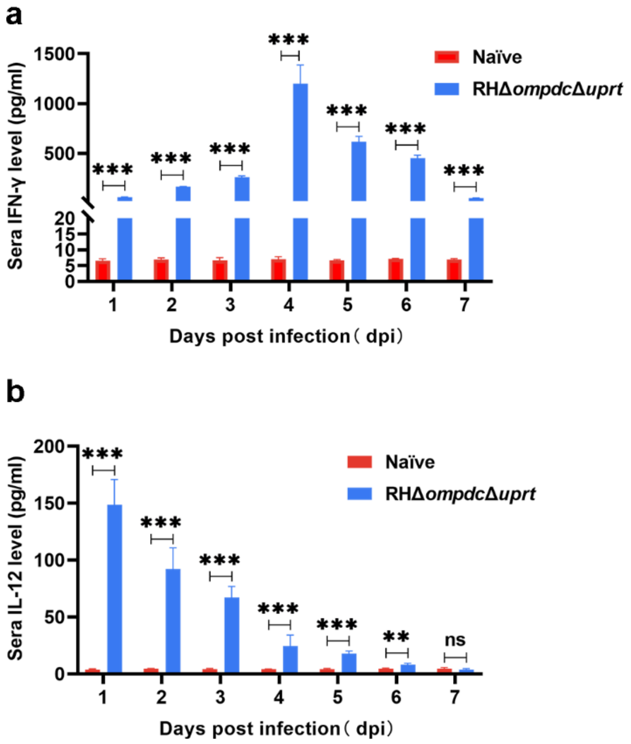

Supplementary Figure4 (Fig.4S). The levels of cytokines in the serum of mice injected with RHΔ*ompdc*Δ*uprt.* The levels of IFN-γ **(a)** and IL-12 **(b)** were measured by flow cytometer. The results are presented as the means ± *SD*. (*n* = 5; ***P* < 0.01, ****P* < 0.001 by Student's *t* test, ns not significant.)
